# Supplementary material for: An Aptamer-Based Proteomic Analysis of Plasma from Cats (Felis catus) with Clinical Feline Infectious Peritonitis
Source: Viruses. 2024 Jan 18;16(1):141. doi: 10.3390/v16010141 (PMC10819688; doi:10.3390/v16010141)
Supplement: Supplementary file 1 [file viruses-16-00141-s001.zip › Supplemental Figures.pdf]

Supplemental Figures:
Figure S1: MAPK signaling
Figure S2: JAK STAT signaling pathway
Figure S3: Natural Killer Cell Mediated Cytotoxicity

Supplemental Tables - a single excel file, tab titles are listed below
Table S1: SOMAscan protein IDs
Table S2: SOMAscan results
Table S3: SOMAscan with prot. & cat IDs
Table S4: t-tests
Table S5: Enrichment Results
Table S6: Enrichment Path. Input List
Table S7: Gene ID duplication cleanup
Table S8: Patient Information
