# Supplementary figures and images for: An Aptamer-Based Proteomic Analysis of Plasma from Cats (Felis catus) with Clinical Feline Infectious Peritonitis
Source: Viruses. 2024 Jan 18;16(1):141. doi: 10.3390/v16010141 (PMC10819688; doi:10.3390/v16010141)

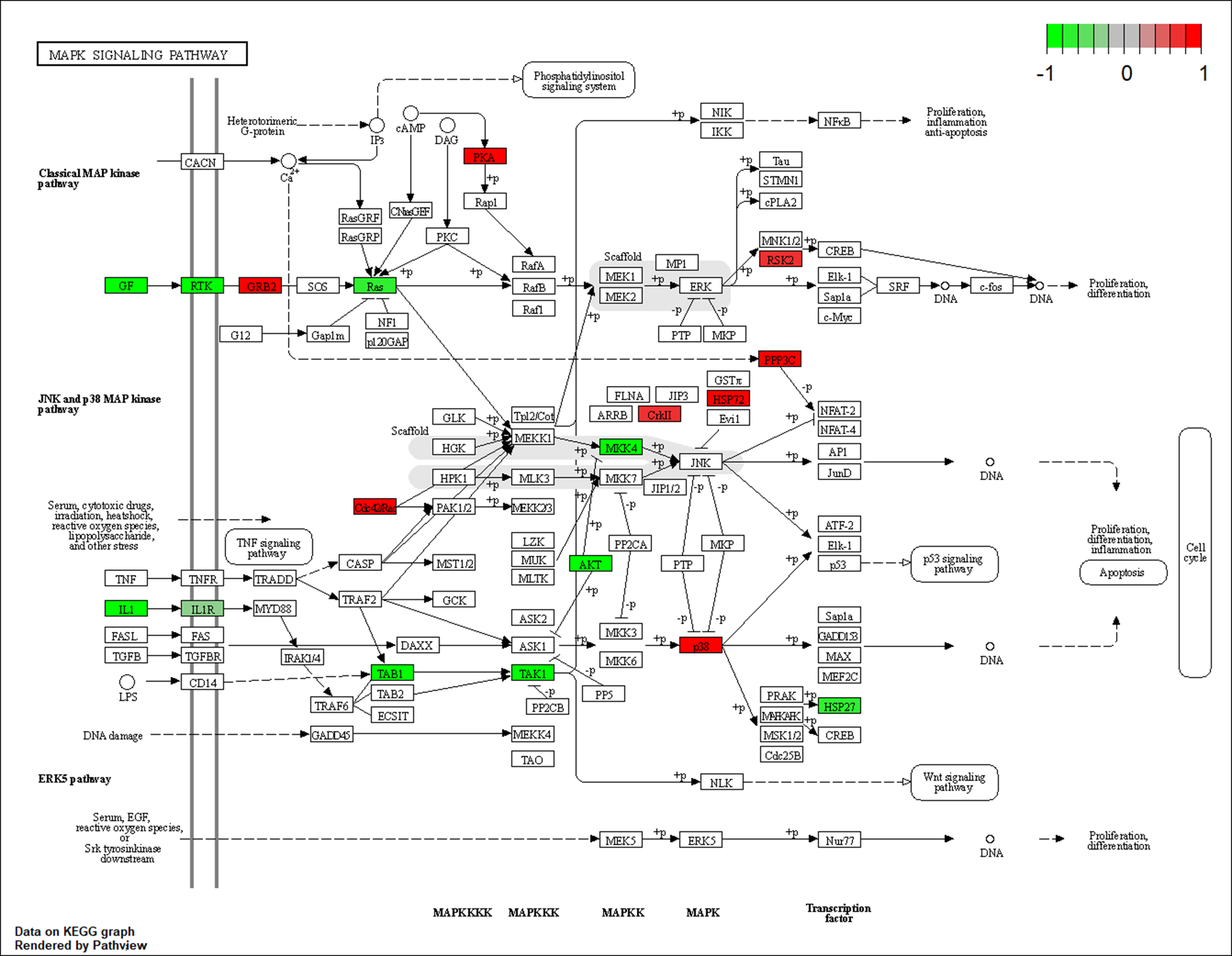

Supplement: Supplementary file 1 [file viruses-16-00141-s001.zip › Figure S1 - MAPK Signaling.tif]

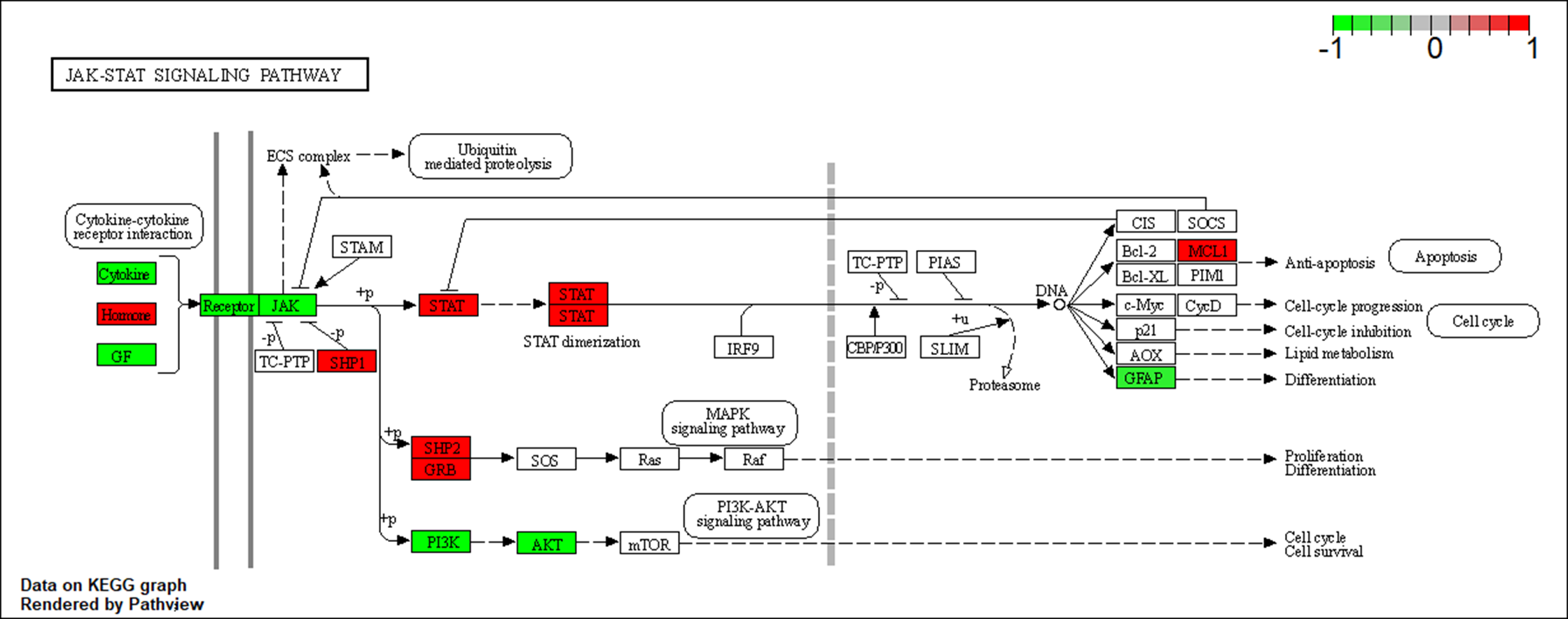

Supplement: Supplementary file 1 [file viruses-16-00141-s001.zip › Figure S2 - JAK STAT signaling pathway.tif]

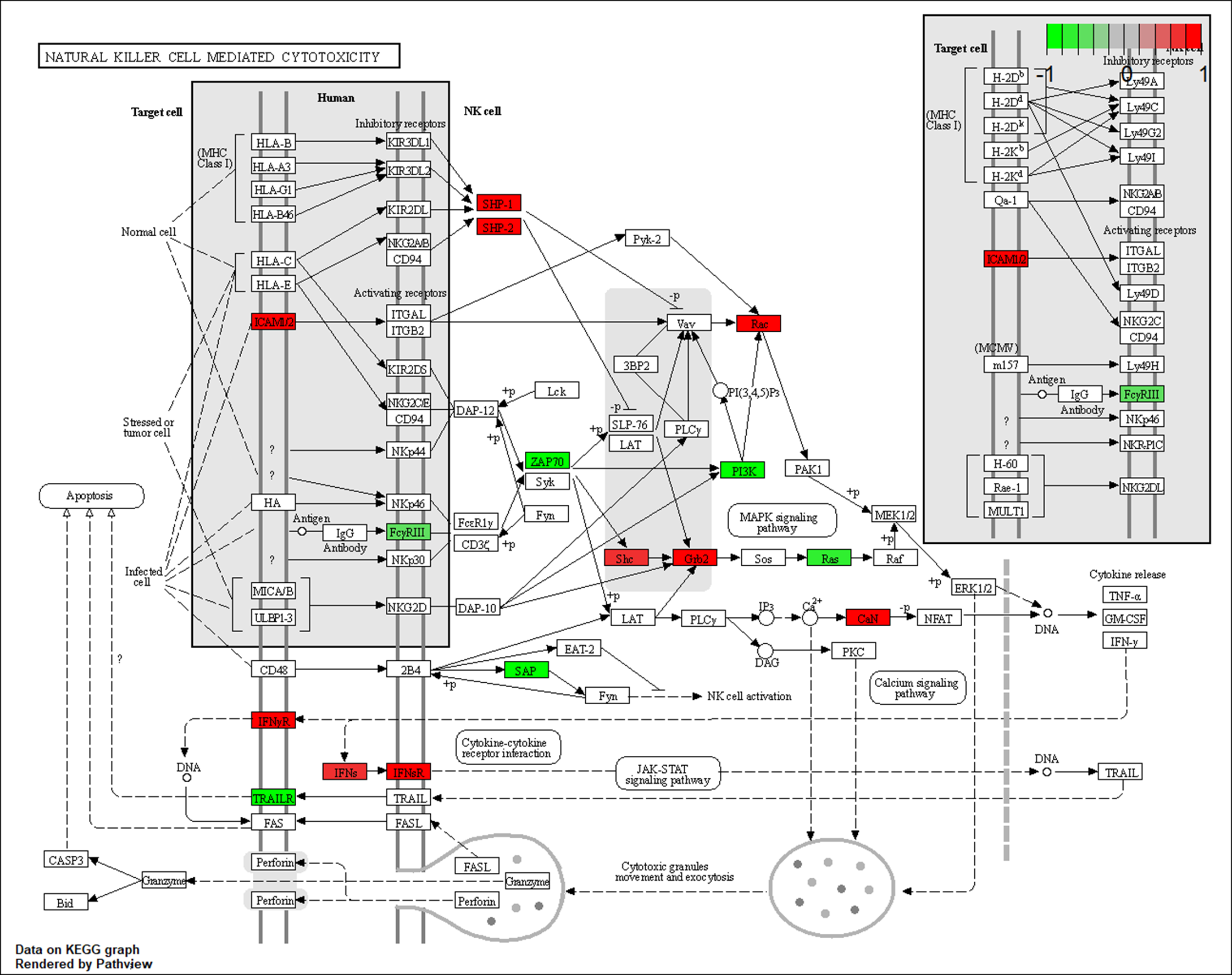

Supplement: Supplementary file 1 [file viruses-16-00141-s001.zip › Figure S3 - Natural Killer Cell Mediated Cytotoxicity.tif]
